# A cross-sectional study to validate an administrative back pain severity classification tool based on the graded chronic pain scale

**DOI:** 10.1038/s41598-022-21422-x

**Published:** 2022-10-08

**Authors:** M. Hochheim, P. Ramm, M. Wunderlich, V. Amelung

**Affiliations:** 1 Institute of Epidemiology, Social Medicine, and Health System Research, Hannover Medical School (MHH), Carl-Neuberg-Straße 1, 30625 Hannover, Germany; 2 Generali Health Solutions GmbH (GHS), Hansaring 40 - 50, 50670 Köln, Germany

**Keywords:** Health services, Chronic pain, Epidemiology, Musculoskeletal system

## Abstract

Treatment of chronic lower back pain (CLBP) should be stratified for best medical and economic outcome. To improve the targeting of potential participants for exclusive therapy offers from payers, Freytag et al. developed a tool to classify back pain chronicity classes (CC) based on claim data. The aim of this study was to evaluate the criterion validity of the model. Administrative claim data and self-reported patient information from 3,506 participants (2014–2021) in a private health insurance health management programme in Germany were used to validate the tool. Sensitivity, specificity, and Matthews’ correlation coefficient (MCC) were calculated comparing the prediction with actual grades based on von Korff’s graded chronic pain scale (GCPS). The secondary outcome was an updated view on direct health care costs (€) of patients with back pain (BP) grouped by GCPS. Results showed a *fair* correlation between predicted CC and actual GCPS grades. A total of 69.7% of all cases were correctly classified. Sensitivity and specificity rates of 54.6 and 76.4% underlined precision. Correlation between CC and GCPS with an MCC of 0.304 also indicated a *fair* relationship between prediction and observation. Cost data could be clearly grouped by GCPS: the higher the grade, the higher the costs and the use of health care. This was the first study to compare the predicted severity of BP using claim data with the actual severity of BP by GCPS. Based on the results, the usage of CC as a single tool to determine who receives CLBP treatment cannot be recommended. CC is a good tool to segment candidates for specific types of intervention in BP. However, it cannot replace a medical screening at the beginning of an intervention, as the rate of false negatives is too high.

*Trial registration* The study was conducted using routinely collected data from an intervention, which was previously evaluated and registered retrospectively in the German Registry of Clinical Trials under DRKS00015463 (04/09/2018). Informed consent and the self-reported questionnaire have remained unchanged since the study and, therefore, are still valid according to the ethics proposal.

## Introduction

Low back pain (LBP) is a prevalent symptom that occurs in all age groups. It is currently ranked as the most common cause of disability worldwide^1^. A prevalence of 7.5% in 2017 indicates that 577 million people around the world have experienced LBP in that year. The lifetime prevalence of LBP is as high as 70–80%, i.e. almost everyone will suffer from back pain (BP) during their lifetime^2–4^. Usually, an exact pathologic cause of LBP cannot be identified, which is why most cases are classified as nonspecific^2,5,6^. LBP is often divided into three stages of chronicity according to the duration of an episode of pain. Acute (< 6 weeks), subacute (6–12 weeks) and chronic (> 12 weeks). Most of those affected recover within the first six weeks. However, 10–40% remain in a state of pain^3,4^.

Treatment of BP depends on chronicity and focuses on different areas. After a short screening for 'red flags', which potentially indicate a specific cause and a more serious aetiology, patients with acute non-specific BP should be generally informed about the pain aetiology, course and likelihood of having LBP and encouraged to remain active^4,7,8^. Due to the natural course of BP, early interventions to prevent progression from acute to chronic state are unnecessary for the majority of patients^9,10^.

Structured physical and psychological interventions are recommended, education on the course of LBP, the neurophysiology of pain, and further encouragement to resume daily activities for patients whose BP is subacute or chronic and those with current acute pain, but at high risk of becoming chronic^4,7,11,12^. There are various approaches that promise a favourable outcome^10,13–16^. These interventions are effective, but also costly and limited in number^16,17^. Furthermore, BP is often not treated according to guidelines and best practices^11,18^. Access to and transitions from primary care to these interventions are still rare. There is also a too frequent and prematureuse of imaging, spinal injection therapies, and surgical procedures in the treatment of chronic lower back pain (CLBP)^7^. In a recently published study, Daniel and colleagues used administrative data from a large statutory health insurance (SHI) in Germany and stated that only 23% of newly diagnosed patients with CLBP receive guideline-based multimodal therapy in the first year of the occurrence^19^. There is still a large gap between evidence and practice in the treatment of BP^11,18^. Multiple reasons for this gap exist^18^. A physician might decide against a guideline-compliant treatment due to social influences. Patients demand a specific diagnosis (leading to imaging) or a quick solution of their issue (injection/surgery). Another factor is the fear overlooking a serious condition, which often leads to imaging to reassure patients. Lastly, the financial framework set by the health system may explain the current gap: it may offer misplaced incentives that make imaging, surgery, and injections more lucrative. Moreover, an inadequate remuneration of a physician's time effort for anamnesis and education may contribute to the gap in LBP care^7,18,20^.

To overcome this gap, the German Federal Joint Committee (G-BA) published basic recommendations for the implementation of a guideline-based disease management programme (DMP) for patients with CLBP in the German Statutory Health Insurance (SHI)^21^. The G-BA has the task of selecting chronic diseases that are suitable for a structured DMP and to determine the content requirements for such programmes more precisely. The implementation process can only start when a disease has been selected by the G-BA^22,23^. However, so far no DMP for CLBP has been implemented^24^. The concrete implementation occurs regionally through different sickness funds of the statutory health insurance system^25^. The contents of back pain-specific DMPs in Germany are subject to negotiations between sickness funds and providers^24^. Each DMP must be approved by the Federal Office for Social Security^26^.

One of the main recommendations of the G-BA is that the CLBP-DMP is offered to patients with at least 12 weeks of persistent pain and a Graded Chronic Pain Scale (GCPS) grade of II or higher, which means that they must be affected by intense BP with mild to severe dysfunction^27,28^.

The SHI is based on a solidarity-competition system. Ninety percent of the total population is insured in the SHI system. Members have the freedom to choose between 97 different sickness funds^29^. To ensure that differences in the structure of insured persons between the sickness funds do not result in unequal competitive opportunities, a risk compensation is in place^22^. As the sickness funds also receive a financial incentive from the statutory risk structure compensation scheme for every participant enrolled in a DMP (currently €145.44)^30^, they are incentivised to maximise the number of participants. Sickness funds and private health insurances can contact insureds to advertise and invite for DMPs. A targeted offer from the payer could lead to greater motivation to participate^31–33^. However, insurance would need to know which clients are not expected to naturally improve their BP to focus their efforts on potential beneficiaries, that is, those likely to receive a medical benefit through an intervention. Following the recommendation for enrolment in a DMP, only clients of GCPS grade II or higher would be contacted in an ideal scenario^21^.

However, selecting CLBP clients from administrative data is not easy, as there is no classification by chronicity in the ICD-10 system^34^. No distinction is made between acute, subacute, and chronic pain. Most BP is simply classified as ´*Low back pain’* (a diagnosis of M54.5)^35^.

To address this deficit, Freytag et al. developed a tool based on routine data from an SHI in Germany with 5.2 million beneficiaries to classify patients with BP before invitation^36^. They performed a secondary data analysis and used the data originally intended for medical settlements to build the classification tool^37^. In order to carry out meaningful health care management with an assessment of the severity of BP they analysed the number of BP-specific ICD-10 diagnoses (M40–M54), information about incapacity to work, opioid intake and psychiatric comorbidities. As a result, they developed a tool to divide patients into three classes of chronicity (CC): 1: *without evidence of chronicity*, 2: *evidence of risk of chronicity, and* 3: *evidence of chronicity*. However, they neither validated their assessment with actual patient feedback or patient-reported results, nor compared it with the self-reported gold standard for assessing the severity of chronic pain in Germany (that is, the Graded Chronic Pain Scale by Von Korff et al./GCPS^27^) so that the criterion validity is lacking^36^.

As the exact content of DMPs is negotiated with providers regionally, health care decision makers have a certain negotiation leeway in terms of cost^25^. Ideally, they would implement and offer an intervention that is dominant in terms of health economics, that is, an improvement in health status on the one hand and a reduction in direct medical costs on the other^38–40^. But since GCPS is currently not collected routinely, providers do not know the grade of their insureds or the average annual costs those insureds produce to treat their back pain condition. Therefore, an estimation of costs and revenues to be expected is hardly possible. Orientation on the scientific literature yields only limited benefit, as so far there are only two studies, which depict the healthcare costs of patients with CLBP by chronicity grades^39,41^, for the German insurance market. The study by Wenig et al.^41^ was conducted in 2005 and only used a postal survey and asked participants insured with SHI with BP (n = 5650) about their use of healthcare in the previous 3 months. An update of healthcare cost due to CLBP, which are not surveyed but calculated based on a claims data base, would help health decision makers in reaching a more informed cost allocation decision.

Therefore, the primary objective of this study was to assess the criterion validity of the classification model. Medically and economically, differences between the two models (GCPS vs. CC) were identified. These were used for the secondary objective of an updated representation of the care costs of CLBP patients in Germany.

## Methods

### Study design and setting

This was a cross-sectional study. For the investigation of these research questions, routine data from the private health insurance (PHI) provider Generali Deutschland Krankenversicherung AG (Generali Germany Health Insurance, formerly known as “Central Krankenversicherung”) was analysed. Since 2014, Generali has been running a 12 month-long primary care biopsychosocial intervention offered proactively to CLBP clients^42,43^. We compared the self-reported GCPS of all participants with the administratively identified CC. As the present research was conducted with routinely collected health data the REporting of studies Conducted using Observational Routinely-collected health Data (RECORD) Statement was used as a guideline to report our results^44^.

### Participants

Study participants were all insured members of Generali, who registered for the intervention against CLBP between July 2014 and March 2021. They were located throughout Germany and had a minimum age of 18. Two ways to register for the intervention existed. The standard way (I) was an invitation sent by the insurance company based on the administrative indication of BP. The minimum invitation requirement was the presence of two diagnoses of ICD-10 in the range of M40 to M54 within the last 12 months. The alternative path (II) was based on the client’s initiative, where they directly requested participation in the health programme (referred to as *self-selected*). For the primary outcome invited persons (I) who enrolled within three months after invitation were used. For the secondary outcome of the cost analysis, participants who proactively requested participation (II) were additionally taken into account.

Excluded from the intervention were individuals with any condition that precluded participation in an intensive physical intervention (e.g., stroke or need for care). The complete exclusion list can be consulted elsewhere^42^.

### Measurement tools

For this study, two data sources were used. Information to calculate CC and its connected variables (e.g., diagnoses, sick-days, opioid use, Charlson’s Comorbidity Index Score), as well as all cost data, was obtained from insurance claims data. The information to calculate the GCPS was obtained through the responses of the participants in the self-administered standardised digital assessment during enrolment. All participants in the health programme underwent this standardised, digital self-assessment procedure consisting of multiple questionnaires before enrolment to ensure that they were eligible for the intervention. It included the GCPS, PHQ-4 sum scores for anxiety and depression^45^, assessments of general health status (first item of the SF-12 questionnaire) as well as information on physical activities and movement pattern. The full list can be found elsewhere^43^.

Completion of the digital self-assessment was mandatory to finish the registration for the intervention. Participants were asked about their current health status to (a) assign the best type of intervention and (b) control individual developments with follow-up measurements. Data management and statistical analyses were performed using the R software^46^ and the packages listed^47–50^.

### Classes of chronicity

For every invited participant the CC was calculated before the invitation^36^. The following routinely collected data from 12 months prior to invitation were taken into account:

- Number of BP-specific ICD-10 diagnoses (M40–M54)
- Incapacity to work due to BP and its duration
- Use of strong opioids (ATC Group: N02A) as an indication of chronic pain and
- Psychiatric ICD-10 F diagnoses: F32*, F33*, F34.1, F34.8, F34.9, F38, F41.2, F45.4, F48.0, F43.20, F43.21, F43.22, F54, F62.80

The three chronicity classes were assigned as follows:

1. Without evidence of chronicity:
  Two diagnoses of M40–M54 and not of CC group 2 or 3.
2. Evidence of the risk of chronicity:
  Two M40–M54 diagnoses combined with less than two opioid prescriptions and either (a) incapacity to work due to a diagnosis of M40 to M54 of less than six weeks or (b) at least two F diagnoses.
3. Evidence of chronicity:
  Two diagnoses of M40–M54 combined with (a) incapacity to work for at least six weeks or (b) at least two opioid prescriptions within six months.

### Graded chronic pain scale

The purpose of the study was to analyse the psychometric properties of the CC classification tool^51^. Currently, there are many different screening tools to assess back pain^52–54^. In Germany, GCPS has become a de facto gold standard for assessing the severity of CLBP, as it is recommended in the national care guideline^8^, the DMP guideline^21^ and used in numerous publications considering the treatment and severity of CLBP^39,42,55–58^. The German version of the GCPS is reliable (Cronbach’s alpha = 0.82) and has a high internal and concurrent validity compared to other staging systems^59^. Therefore, GCPS was used as a reference value for the classification of the severity of back pain and compared with CC. Information required to calculate Graded Chronic Pain Scale (GCPS) scores^27,28^ were extracted from the digital self-assessment.

The GCPS questionnaire uses three items, rated on a scale of 0 to 10, to obtain a pain intensity score, calculated as the average of 0 to 10 ratings of “pain right now”, “average pain” and “worst pain” (both in the last six months) multiplied by ten to yield a score between 0 and 100. Similarly, the GCPS uses four items to measure disability in terms of the impact of a disease on daily, social and work activities (disability score) and the number of disability days (all questioning about the last 6 months). A disability score is calculated in the same way as the pain intensity score (0–100). Additionally, disability days and score are categorised into groups and combined to total disability points. The GCPS is formed from combining the pain intensity score and the disability points. Based on their answers to the seven test items, each participant was assigned an overall GCPS grade as follows: Grade I—low to moderate disability, low intensity, Grade II—low to moderate disability, high intensity, Grade III—high disability, moderately limiting, and Grade IV—high disability, severely limiting^27^.

### Participant characteristics potentially associated with the grade of chronic BP

The self-assessment data from all health programme participants were used in combination with insurance provider claims data to answer the research question at hand. Demographic (sex, age, job status), health system utilisation (total health costs, back pain-specific costs, number of ICD-10 back pain-related [M*] and anxiety and depression-related [F*] diagnoses), and comorbidity type and severity data (Charlson's Comorbidity Index Score (CCI)) were extracted from the health insurance system’s population and claims database from 2013 to 2021. Possible psychological comorbidities (PHQ-4 score and its subscales)^45^ and direct effects of BP (daily impairment, average level of pain, number of days restricted in everyday activities within the last six months) were extracted from the digital self-assessment^43^. These variables were descriptively compared across CC respective GCPS grades.

### Assessing criterion validity (CC)

The primary outcome was the criterion validity of the CC, i.e., the degree to which the classification model of chronicity classes of BP using claim data developed by Freytag and colleagues is an adequate reflection of the gold standard^60^. The predicted (CC) was compared with the actual chronicity grade (GCPS) for all invited participants in the intervention. To compare the four-level GCPS with the three-level CC, the GCPS needed to be reduced by one grade. GCPS grades I and II were combined and compared with *CC 1—“without evidence of chronicity”.* GCPS grade III was compared with CC 2—“*Evidence of risk of chronicity”* and GCPS grade IV with *CC 3*—*“evidence of chronicity”*.

In a first step, the correlation between CC and newly categorised GCPS was evaluated using Spearman’s rho rank correlation coefficient with 95% confidence intervals (CI). The strength of the correlation was interpreted as *weak* (rho < 0.1), *modest* (rho 0.1–0.3), *moderate* (rho 0.31–0.5), *strong* (rho 0.51–0.8) or *very strong* (rho > 0.8)^61^. The second step included the evaluation of the agreement between CC and the GCPS categorised using Cohen’s weighted Kappa. The agreement was interpreted as *poor* (Kappa < 0.2), *fair* (Kappa 0.21–0.4), *moderate* (Kappa 0.41–0.6), *substantial* (Kappa 0.61–0.8) or *almost perfect* (Kappa 0.81–1)^62^.

According to the COnsensus-based Standards for the selection of health Measurement Instruments (COSMIN) checklist for patient-reported outcome measurement instruments to assess the criterion validity, the scores should be dichotomous or continuous^63,64^. Therefore, GCPS and CC were dichotomised in the cases of severe and non-severe BP. Grades I and II were previously defined as *functional* chronic pain, and Grades III and IV as *non-functional* chronic pain^27^. In order to allow easier comparability and interpretation, GCPS grades I and II, which were already summarised, were relabelled as *non-severe* and grades III to IV as *severe* cases. CC class 1 and 2 equally as *non-severe*, and CC 3 as *severe* BP cases and presented in a 2 × 2 confusion matrix.

The confusion matrix assigned the chronicity class of each health programme participant with its predicted class (severe BP or non-severe BP). As a result, every sample belonged to one of the following four classes:

- *True positives (TP)* were actual cases of severe BP that were correctly predicted as severe.
- *True negative (TN)* were actual cases of non-severe BP that were correctly predicted as non-severe.
- *False positive (FP)* were actual cases of non-severe BP that were wrongly predicted as severe.
- *False negative (FN)* were actual severe BP cases that were incorrectly predicted as non-severe.

Sensitivity (i.e. the proportion of participants with severe BP that were correctly classified by the model), specificity (i.e. the proportion of participants without severe BP correctly classified as not having severe BP by the model) and Matthews correlation coefficient (MCC)^65^ (i.e. the correlation between actual and predicted severity grades) were estimated to evaluate the performance of the model. MCC was chosen instead of accuracy and F_1_ score, as it is more reliable taking into account all four categories of the confusion matrix^66^. As MCC is a discrete case of Pearson Correlation Coefficient, the strength of the correlation was interpreted equally, meaning: *very weak relation* (MCC 0.01–0.29), *fair relation* (MCC 0.3–0.59), *moderately strong relation* (MCC 0.6–0.79) or *very strong relation* (MCC >  = 0.8)^67^. Cohen’s weighted Kappa was again estimated as a concordance statistic.

$$Sensitivity = { }\frac{{Severe\,BP\,cases\,correctly\,identified\,using\,claims\,data}}{{Total\,severe\,BP\,cases\,in\,MBR}}{ } \times { }100{ }$$

$$Specificity = { }\frac{{Non\,severe\,BP\,cases\,correctly\,identified\,using\,claims\,data}}{{Total\,non\,severe\,BP\,cases\,in\,MBR}}{ } \times { }100{ }$$

$$MCC = { }\frac{{TP \times { }TN - FP{ } \times { }FN}}{{\sqrt {\left( {TP + FP} \right) \times { }\left( {TP + FN} \right) \times { }\left( {TN + FP} \right) \times { }\left( {TN + FN} \right)} }}{ }$$

### Sensitivity analysis

Not all participants held a daily sickness benefit insurance policy in addition to their regular at Generali. Most of the participants are likely to have been insured against sick leave by another provider. However, no information on insurance status was available. Therefore, in contrast to the SHI system, there was no general incentive for the insured to report incapacity to work to Generali. Since days of incapacity to work play an important role in the calculation of CC, the insurance status of the daily sickness allowance of the insured was regarded as a possible confounder and was analysed separately in a sensitivity analysis. It was assumed that those insured against sick leave at Generali also reported absence.

### Cost of CLBP care

The secondary outcome was an updated representation of the costs of CLBP care in the German PHI setting. Overall health costs and hospital costs specific to BP, as well as outpatient costs, were considered in the last 12 months prior to enrolment. Costs were descriptively compared across CC respective GCPS grades.

Included were costs from the following areas: General hospital services, general practitioner and specialist care, medicines, remedies, alternative practitioners (e.g., chiropractor), aids, and private medical treatment. Additional elective services (e.g., one or two-bedroom supplement) and the entire costs of dental care treatment were excluded.

### Cost data analysis

In a PHI setting, the reimbursement procedure follows the principle of refund of expenses, i.e., the clients pay the health care bill in advance, submit the bill afterwards to their insurance company, and receive the reimbursement according to the insurance tariff concluded from it. The reimbursement of health care bills depends on the respective tariff. The study population consisted of fully insured participants with different levels of deductible, as well as policyholders eligible for governmental aid. Therefore, the cost component was defined as the total amount of the bill instead of the amount of refund paid. Thus, the actual costs were compared with each other without taking into account which payer (health insurance, subsidy, or individual supplementary) reimbursed the costs. As the costs were presented for a period of 12 months, no discounting was carried out. All costs were converted to 2020 euros (€) using consumer price indices.

As healthcare care costs tend to be heavily skewed and right-tailed^68^, a truncated mean was also calculated in addition to the average costs per category. For this, all upper outliers (high-cost cases) were calculated using Tukey’s method with 1.5 * interquartile range (IQR)^69^. Low-cost cases were defined as participants who did not submit an invoice from the presented area in the last 12 months prior to enrolment.

### Materials

The study used routinely collected data from an intervention, which was previously evaluated and retrospectively registered in the German Registry of Clinical Trials under DRKS00015463 (04/09/2018). The consent to participate and the self-reported questionnaire have remained unchanged since the study and are therefore still valid according to the ethics proposal. The independent research ethics committee of the University of Lübeck gave approval for the original evaluation study (Re.-No.14 –249, dated 20/11/2014). As the participants consented to the usage of the data for further analysis, no new ethic vote was sought for the present analysis. This was in accordance with national legislation (e.g., SGB V, paragraph 303e). All procedures performed in the present study were in accordance with the 1964 Helsinki declaration and its later amendments. Written informed consent was obtained from all study participants. A written agreement to use and process the raw data was concluded between Generali Health Solutions (GHS) and Generali Deutschland Krankenversicherung AG. This also explicitly includes the right to use the data for research purposes. As employees of GHS, MH, PR and MW therefore have access to the raw data and are allowed to use it for research purposes.

## Results

### Study size

Different samples were required to answer the two research questions. The selection criteria are shown in Table 1. The study population consisted of 3629 participants for whom the GCPS grade was available at enrolment. As the data was provided by a PHI, there were participants with an individual yearly threshold of costs before payment of expenses (deductible). Insurees with a fixed deductible usually only hand in their invoices of a year if they exceed that amount. To reduce the potential bias introduced by the tariff, 123 participants who did not provide any invoice in the 12 months prior to enrolment (annual average invoices = 27) were excluded.

**Table 1:** Data preparation processes for selection of study population.

Data processing steps showing the number of participants	Overall	Used in
Study size	3629	
Exclusion of participants without any billing invoice available	3506	Research question II
Enrolment after invitation by insurance	2722	
Enrolment within 90 days after invitation	2396	Research question I
Enrolment within 90 days after invitation plus insured against sick leave	1114	Sensitivity analysis

To answer the first research question of the criterion validity, all participants who enrolled in the standard way (n = 2722) were taken into account. The time between the initial invitation and enrolment was calculated. As the CC was only available on the date of invitation, participants who took more than 90 days to register were excluded (n = 326) in order to rule out the temporal effect and therefore possible changes of the CC. The final group size for the first research question was 2,396.

To estimate the cost of CLBP participants signed up on their own initiative were additionally considered (n = 872). The size of the group used to answer the second research question increased to 3506.

### Participants

Characteristics of the study population are presented in Table 2. The mean age of the participants was 54.74 years, while 65.9% were of male and 34.1% of female sex. The mean CCI was 0.8, indicating a population in a healthy state. This was confirmed at the assessment. More than two-thirds self-reported an overall health status of “moderate” or better. The average sum score of PHQ-4 was 2.82. The PHQ-4 subscales averaged 1.56 in depression and 1.26 in anxiety. The average intensity of pain was 4.48 and the average disability was 3.98, while both were assessed using the GCPS questionnaire. Most of the participants reported having less than 14 days of disability due to their BP within the last six months. Almost half (46.1%) of the study population was insured against sick leave at this provider. In total, 8.7% made a claim for sick leave due to BP.

**Table 2:** Characteristics of study participants based on Graded Chronic Pain Grades.

	Overall	GCPS I	GCPS II	GCPS III	GCPS IV	*p*
N	3506	1499 (42.8)	867 (24.7)	622 (17.7)	518 (14.8)	
Sex = Female (%)	1196 (34.1)	477 (31.8)	328 (37.8)	204 (32.8)	187 (36.1)	0.016
Age (mean (SD))	54.73 (9.52)	54.63 (9.56)	54.00 (9.59)	55.55 (9.44)	55.28 (9.30)	0.009
CCI–score (mean (SD))	0.80 (1.41)	0.71 (1.36)	0.72 (1.29)	0.88 (1.46)	1.06 (1.63)	< 0.001
**Overall health (%)**						< 0.001
Very good	49 (1.4)	41 (2.7)	2 (0.2)	2 (0.3)	4 (0.8)	
Good	505 (14.4)	340 (22.7)	105 (12.1)	41 (6.6)	19 (3.7)	
Moderate	1832 (52.3)	916 (61.1)	490 (56.5)	279 (44.9)	147 (28.4)	
Bad	970 (27.7)	200 (13.3)	250 (28.8)	261 (42.0)	259 (50.0)	
Very bad	150 (4.3)	2 (0.1)	20 (2.3)	39 (6.3)	89 (17.2)	
PHQ-4 score (mean (SD))	2.82 (2.56)	1.76 (1.83)	2.75 (2.18)	3.79 (2.65)	4.83 (3.14)	< 0.001
PHQ-4 subscale depression	1.56 (1.41)	0.94 (1.01)	1.53 (1.23)	2.12 (1.43)	2.70 (1.63)	< 0.001
PHQ-4 subscale anxiety	1.26 (1.37)	0.82 (1.04)	1.22 (1.25)	1.67 (1.46)	2.12 (1.71)	< 0.001
Average pain intensity within the last six months (mean (SD))	4.48 (1.95)	2.95 (1.32)	5.33 (1.26)	5.52 (1.63)	6.23 (1.62)	< 0.001
Average disability within the last six months (mean (SD))	3.99 (2.44)	2.17 (1.58)	4.03 (1.64)	5.62 (1.70)	7.18 (1.47)	< 0.001
**Days disabled (%)**						< 0.001
0–6 days	2160 (61.6)	1353 (90.3)	710 (81.9)	97 (15.6)	0 (0.0)	
07–14 days	445 (12.7)	121 (8.1)	147 (17.0)	176 (28.3)	1 (0.2)	
15–30 days	398 (11.3)	22 (1.5)	10 (1.2)	268 (43.1)	98 (18.9)	
31–180 days	503 (14.3)	3 (0.2)	0 (0.0)	81 (13.0)	419 (80.9)	

Definition of GCPS: *Grade I* Low disability-low intensity, *Grade II* Low disability-high intensity, *Grade III* High disability-moderately limiting, *Grade IV* High disability-severely limiting.

A clear division of the characteristics by the GCPS could be observed. There was a clear negative correlation between GCPS and health status. The higher the grade, the lower the health indicators. This was also true for variables, which were not used to calculate the GCPS (i.e., PHQ-4, general health, CCI and sick leave due to BP).

Most of the participants were grouped in GCPS Grades I (42.8%) or II (24.7%). Grade III (17.7%) and IV (14.8%) made up one third of the participants. Characteristics were also divided by CC in comparison (see Supplementary Table S1 online). It could be observed that CC 2 (evidence of risk of chronicity) reported on average a higher PHQ-4 than CC 1, CC 3 or self-selected participants.

**Figure 1:**
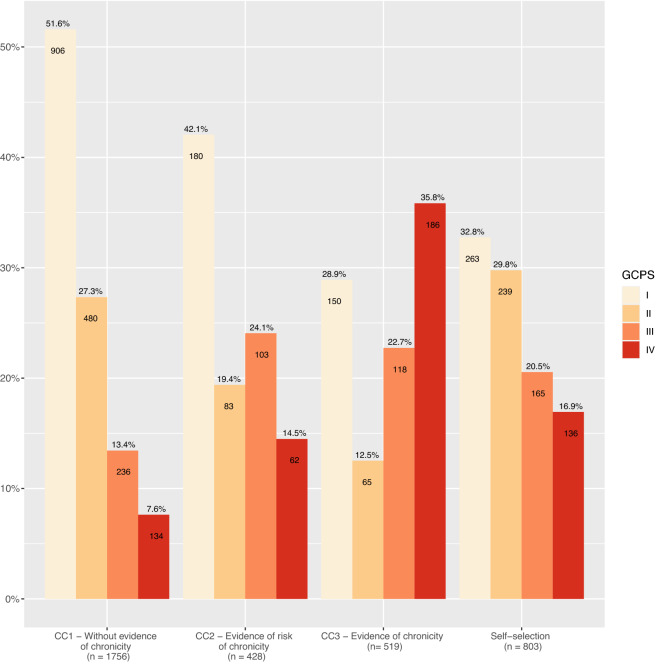
Comparison of actual BP severity with prediction. Comparison of GCPS at baseline with prediction from claims data algorithm according to Freytag et al.

Figure 1 shows the distribution of the GCPS grades per CC. More than every second participant (51.6%) with CC 1 prediction belongs to GCPS grade I, 27.3% to GCPS II, 13.4% to GCPS III and 7.6% to GCPS IV. Most of the participants predicted to belong to CC 2 are grouped into non-severe BP (GCPS grade I: 42.1%, II: 19.4%). More than half of the predicted members of CC 3 belong to the severe BP group (GCPS grade III: 22.7%, grade IV: 35.8%). Self-selected participants are distributed across all categories (GCPS grade I: 32.8%, II: 29.8%, III: 20.5%, IV: 16.9%).

### Criterion validity of chronicity classes

The primary outcome was to analyse the criterion validity of the algorithm to define chronicity classes according to Freytag et al.^36^. The predicted CC was compared with the actual grade of chronicity (GCPS) for all invited participants. A total of 2396 participants were classified into one of the three classes (*chronic, risk of chronification, or non-chronic BP*) (see Supplementary Table S2 online). With a value of 0.343 (95% CI: 0.307–0.377, *p* < 0.001) in Spearman’s rho, the correlation between the classes could be classified as *moderate*. A weighted Kappa of 0,307 (CI 0.268–0.346) indicated a *fair* agreement between CC and GCPS.

Additionally, GCPS and CC were dichotomised in severe or non-severe BP cases. Table 3 shows the confusion matrix, which matches the assigned CC of each health programme participant with its predicted class. The results show a sensitivity of 54.6% and a specificity of 76.4%. In total, 69.7% were correctly predicted. With an MCC of 0.304, the strength of correlation was classified as *fair*. This was in agreement with Cohen’s weighted Kappa of 0.304 (95% CI: 0.260–0.348), which also indicated a *fair* agreement between CC and GCPS.

**Table 3:** Evaluation of the predicted severity of BP with self-reported GCPS grades.

Predicted severity category of BP	Observed BP severity category		
	Severe (GCPS III & IV)	Non-severe (GCPS I & II)	Total
Severe (CC 3)	TP: 401	FP: 392	793
Non-severe (CC 1&2)	FN: 333	TN: 1270	1603
Total	734	1662	2396
Sensitivity	54.6%		
Specificity	76.4%		
Correctly predicted	69.7%		
MCC	0.304		
Cohen’s weighted Kappa	0.304 (95% CI: 0.260–0.348)		

*GCPS* Graded chronic pain grade^27^ based on a self-questionnaire at enrolment, *CC* Chronicity class^36^ based on administrative claim data, *TP* True positive were actual severe BP cases that were correctly predicted as severe, *TN* True negative were actual non-severe BP cases that were correctly predicted as non-severe, *FP* False positive were actual non-severe cases of BP that were wrongly predicted as severe, *FN* False negative were actual severe BP cases that were incorrectly predicted as non-severe.

### Sensitivity analysis

The capacity to work played a pivotal role in the calculation of CC. Only 46.1% of the study population were insured against sick leave at this provider, which meant that only information on working ability was available for that subpopulation. To exclude the possibility of confounding due to insurance status, a sensitivity analysis was performed that included only participants who were insured against sick leave (n = 1114). In a similar fashion as shown above, a 3 × 3 confusion matrix (see Supplementary Table S3 online) was created and afterwards dichotomised. Spearman’s rho (0.405 (0.355–0.453, *p* < 0.001)) and Cohen’s weighted Kappa (0.358 (CI 0.298–0.418)) were increased. The 2 × 2 confusion matrix (Table 4) shows that sensitivity has increased to 63.9%, while specificity has decreased slightly to 73.4%. Overall, 70.7% of all cases were correctly predicted. An MCC of 0.348 and a weighted Kappa of 0.341 also indicated a *fair* relationship between prediction and observation of the severity of BP for the subgroup.

**Table 4:** Sensitivity analysis with participants insured against sick leave.

Predicted severity category of BP	Observed BP severity category		
	Severe (GCPS III & IV)	Non-severe (GCPS I & II)	Total
Severe (CC 3)	TP: 202	FP: 212	414
Non-severe (CC 1&2)	FN: 114	TN: 586	700
Total	316	798	1114
Sensitivity	63.9%		
Specificity	73.4%		
Correctly predicted	70.7%		
MCC	0.348		
Cohen’s weighted Kappa	0.341 (95% CI: 0.275–0.408)		

*GCPS* Graded chronic pain grade^27^ based on a self-questionnaire at enrolment, *CC* Chronicity class^36^ based on administrative claim data, *TP* True positive were actual severe BP cases that were correctly predicted as severe, *TN* True negative were actual non-severe BP cases that were correctly predicted as non-severe, *FP* False positive were actual non-severe cases of BP that were wrongly predicted as severe, *FN* False negative were actual severe BP cases that were incorrectly predicted as non-severe.

### Health care costs of CLBP

The secondary outcome was an updated representation of the costs of CLBP care in the German PHI setting. Overall health costs and BP-specific inpatient, as well as outpatient costs were presented in the last 12 months before enrolment. Costs were descriptively compared across CC respective GCPS grades of participants who were invited by the insurance or took part upon self-selection.

Table 5 presents the cost data for the 3506 participants analysed. The pattern seen in the general characteristics of the study population could also be identified in the health care use and cost information. A clear linear relationship could be observed between the GCPS grade and the variables presented. Healthcare usage and costs increased with increasing GCPS grades. The overall average of total direct health costs was €7,279.78. The 1499 participants with GCPS grade I had a mean cost of €5,967.94, while the 518 participants with GCPS grade IV at enrolment had a mean cost of €10,619.29. The average cost of BP specifics for the overall group was €1,082.13. Participants with GCPS grade IV (€2,312.12) had more than three times higher costs than participants with GCPS grade I (€650.53). The truncated mean of BP-specific costs was €751.73 for the overall group. The cost range between the mean of the participants in GCPS I (€618.80) and IV (€1,051.59) was considerably smaller. Skewness was reduced from 7.2 in the mean to 1.1 in the truncated mean. Cost differences could be explained mainly by high-cost cases. The distribution of high-cost cases varied significantly between groups: The GCPS grade I group showed 3.9% and group IV 24.9% high-cost cases.

**Table 5:** Healthcare use and costs during the 12 months before enrolment according to GCPS.

	Overall	GCPS I	GCPS II	GCPS III	GCPS IV	*p*	Skew
N (%)	3506	1499 (42.8)	867 (24.7)	622 (17.7)	518 (14.8)		
Insured against sick-leave	1616 (46.1)	744 (49.6)	396 (45.7)	260 (41.8)	216 (41.7)	0.001	
Sick-leave due to BP (% of insured against sick-leave)	305 (18.9)	67 (9)	35 (8.8)	72 (27.7)	131 (60.6)	< 0.001	
F-Diagnosis available (%)	644 (18.4)	209 (13.9)	135 (15.6)	152 (24.4)	148 (28.6)	< 0.001	
Amount of ICD-10 F- diagnoses (mean (SD))	1.42 (4.84)	0.95 (3.63)	1.16 (4.55)	1.90 (5.81)	2.60 (6.56)	< 0.001	5.9
Amount of ICD-10 M- Diagnoses (mean (SD))	4.11 (5.67)	2.70 (4.06)	3.45 (4.11)	5.16 (6.66)	8.04 (8.07)	< 0.001	3.0
**Total health** Cost € (mean (SD))	7279.78 (8040.56)	5967.94 (6579.83)	6570.73 (6646.93)	8648.47 (10,151.72)	10,619.29 (9787.68)	< 0.001	3.6
High-cost cases (%)	252 (7.2)	75 (5.0)	48 (5.5)	57 (9.2)	72 (13.9)	< 0.001	
**BP total** Cost € (mean (SD))	1082.13 (2298.10)	650.53 (1531.07)	875.17 (1348.64)	1378.91 (2711.49)	2321.12 (3857.27)	< 0.001	7.2
BP truncated Cost € (mean (SD))	751.73 (670.23)	618.80 (617.22)	733.09 (634.29)	851.71 (682.29)	1051.59 (754.24)	< 0.001	1.1
High-cost cases (%)	325 (9.3)	58 (3.9)	70 (8.1)	68 (10.9)	129 (24.9)	< 0.001	
Low-cost cases (%)	851 (24.3)	475 (31.7)	194 (22.4)	111 (17.8)	71 (13.7)	< 0.001	
**BP inpatient** Cost–€ (mean (SD))	340.70 (1822.82)	160.56 (1146.28)	157.26 (834.00)	463.77 (2114.65)	1021.22 (3398.49)	< 0.001	10.5
High-Cost cases (%)	256 (7.3)	54 (3.6)	44 (5.1)	57 (9.2)	101 (19.5)	< 0.001	
Low-Cost cases (%)	3250 (92.7)	1446 (96.4)	823 (94.9)	565 (90.8)	417 (80.5)	< 0.001	
**BP outpatient** Cost–€ (mean (SD))	741.43 (1047.45)	489.97 (818.02)	717.91 (927.41)	915.14 (1176.70)	1299.90 (1364.68)	< 0.001	2.7
High-cost cases (%)	216 (6.2)	41 (2.7)	52 (6.0)	45 (7.2)	78 (15.1)	< 0.001	
Low-cost cases (%)	863 (24.6)	480 (32.0)	197 (22.7)	111 (17.8)	75 (14.5)	< 0.001	

Definition of GCPS: *Grade I* Low disability-low intensity, *Grade II* Low disability-high intensity, *Grade III* High disability-moderately limiting, *Grade IV* High disability-severely limiting.

*High-cost cases* were calculated using the Tukey method with 1.5 * IQR^69^.

*Truncated mean* Exclusion of high-cost cases and cases who did not submit a BP invoice in the last 12 months prior to enrolment.

Similarly, the number of low-cost cases (i.e., participants who did not submit a BP invoice in the last 12 months prior to enrolment) showed a clear negative trend in combination with GCPS. In total, 851 participants did not submit a BP-related invoice in the last 12 months. Almost one third of those belong to GCPS grade I (31.7%) and 13.7% belong to GCPS grade IV.

A grouping of the costs was also done by the CC (see Supplementary Table S4 online). It could be seen that CC 3 is highly comparable with GCPS grade IV. The average total health costs for CC 3, which involved 519 participants, were €10,277.41. BP specific costs amount to €2,453.11 on average. Of the applicable sick leave participants, 69.5% had to call in sick due to BP in the last 12 months.

## Discussion

### Summary

This study used administrative claim data and self-reported patient information to analyse the criterion validity of a claim-based algorithm (CC) to identify the severity of CLBP. A functioning algorithm would allow payers to select and invite participants for targeted and expensive treatment programs without the need for additional screening. The results showed a *fair* correlation between the predicted CC and the actual GCPS grades. A total of 69.7% of all cases were correctly classified. Sensitivity and specificity rates of 54.6 and 76.4% underlined the precision of the prediction. A sensitivity analysis with participants insured against sick leave showed similar results. The correlation between CC and GCPS with an MCC of 0.348 and a weighted Kappa of 0.341 also indicated a *fair* relationship between prediction and observation of the severity of BP for the subgroup.

Cost data could be clearly grouped by GCPS grades. It could be stated that the higher the grade, the higher the cost and usage of health care. Overall, the average total direct health cost was €7,279.78. Participants with GCPS grade I had mean costs of €5,967.94, while participants with GCPS grade IV had mean costs of €10,619.29. The average BP specific cost for the overall group was €1,082.13. Participants with GCPS grade IV (€2,312.12) had more than three times higher BP specific costs than participants with GCPS grade I (€650.53).

### Limitations

Limitations to the study relate to the data used (I), the outcome (II) and the comparability of GCPS and CC (III).

The administrative data used had the primary purpose of settling claims and were granted by a PHI, who in general have the freedom to implement and offer health programmes without any restrictions due to national regulations. However, PHI data do not include all health-related billing data. Tariff-related peculiarities (e.g., deductibles and co-payments) mean that in practice not all medical invoices are submitted^70^. With the exclusion of participants with no invoices submitted in the last 12 months, possible tariff biases were reduced. But still, only a minority of participants had a daily sickness benefit insurance with this provider. It was likely that there was an underreport of sick leave. Sensitivity analysis focused on participants who were insured against sick leave and showed an improvement in the strength of correlation. However, it could be the case that the algorithm is better suited for a sickness fund where complete information about sick leave for all participants would be available (e.g., the German SHI). In further research, participants should be asked about sick leave directly to cross-validate claim data.

The second limitation was the reduction of classes in the outcome of the criterion validity. The original GCPS had four grades, while CC had only three grades. Therefore, GCPS grades I and II were combined to reduce the amount to three grades. Taking into account cost information and demographic characteristics, it could be stated that this was a legitimate operation, as characteristics between I and II only differed slightly. A dichotomisation of the GCPS in severe and non-severe BP cases was also unproblematic, as this is inherently contained in the classification, which is separating grades I/II and III/IV by disability into functional and non-functional chronic pain. A dichotomisation of the three CC classes could prove to be difficult, as CC 2 was between chronic and non-chronic. However, confusion matrices were run for 3 × 3 and 2 × 2 comparisons, and the results differed only marginally. The strength of the relationship remained in the range of *a fair correlation*, so a reduction to two categories did not influence the overall results.

It should be noted that GCPS is a self-reported instrument that provides information on the severity of CLBP using pain intensity and disability. The CC, on the other hand, is administratively calculated and tries to use routinely collected medical data to assess the risk of chronification. A comparison with CC and other screening tools to predict the development of CLBP (such as the STarT Back Screening Tool or the Örebro Musculoskeletal Pain Questionnaire) could produce different results^53^. Since GCPS is used predominantly in Germany and is recommended to be used to select patients for the intervention and as the CC can also be used for this purpose, we believe that it is still important to compare CC with GCPS. However, we recommend that our findings should be further validated with a comparison between CC and other screening tools.

### Bias

The data routinely collected did not provide a potential source of bias. For the data collected within the standardised, self-administered questionnaire, there were two potential sources of a) recall bias and b) demand characteristics. Recall bias was possible since the GCPS was calculated using the development of BP within the last six months. However, GCPS is generally widely used to assess CLBP^9,39,56,71^ but also other types of anatomically defined pain conditions^72,73^. It has been validated several times^74,75^ and is an internationally recognised tool in self-administered pain assessment^76^ so that a possible effect of recall bias was neglected.

A second possible source of bias was demand characteristics^77^, i.e., that respondents answered the questionnaire tactically to receive the most comprehensible care possible. However, participants were asked to answer truthfully to receive an intervention tailored to their individual needs. Since all participants were pain patients who volunteered for the intervention, which is always free of charge, it could be assumed that their answers were rather accurate. Furthermore, the specific steering logic was not mentioned in writing. Therefore, a bias due to demand characteristics appeared also to be unlikely.

Overall, it has to be stated that a possible selection bias exists. As we used only routinely collected data, we do not have any information about the GCPS of invited insureds who did not respond to the invitation. Potentially, this could mean that there is a difference between GCPS of responders and nonresponders. It may be prudent to revalidate in a sample not tied to participation in a health programme.

### Interpretation

To our knowledge, this was the first study to compare the severity predicted for BP by claims data with the actual severity of BP by GCPS. In other types of disease, predictive models from administrative data were often used to estimate the severity of the disease. Studies that predict the severity of asthma^78^, cancer^79^, COPD^80^ or stroke^81^ identified comparable levels of performance. A correct classification of 70% appears to be a reasonable accuracy to identify the severity of BP. Based on the findings of this study, the use of CC as a single tool is not recommended to determine who is treated against CLBP at what intensity. A sensitivity of 54.6% means that almost half of the participants who are experiencing severe consequences based on GCPS would not be selected by the model. Furthermore, the preferences and personal life circumstances of insured persons should be considered to a reasonable extent when selecting the appropriate program component. When creating further classification algorithms, future research should include a criterion validity assessment in the validation study. Only then is it possible to actually determine whether the algorithm identified also performs well in practice.

In this study, it was also shown that participants with high GCPS grades not only suffer the most, but also cause the highest costs. In previous studies, it was clearly established that patients, especially those with high GCPS grades, benefit from long-term multimodal interventions^39,42,56^. The payer therefore has an interest in trying to focus on this subgroup to reach a cost-effective intervention. The use of CC by Freytag et al. could help select the type of intervention offered proactively to the individual by the payer. As GCPS was significantly differently distributed, it seems likely that the participants have different needs for the intensity/content of the health programme. Using the CC could be a next step to a patient tailored treatment and communication approach^33,82^. If the invitees agree to participate, then the algorithm-specific suggestion would need to be validated by the actual need reported by the patient with a clinical screening item such as STarT Back or GCPS before the appropriate intervention is assigned^83^. More research is needed to identify the best communication to increase participation rates.

The second outcome of the study was an updated view of direct healthcare costs for patients with CLBP. For the German insurance market in which the study was conducted, there were two studies that depicted the healthcare costs of CLBP patients by chronicity levels^39,41^. Wenig et al.^41^ estimated that a patient with CLBP would create on average direct costs of €612.50. They found that the most influential predictor of high costs was a high grade of GCPS. Participants with GCPS grades IV (€7,115.7) were said to have more than 17 times total costs (direct and indirect BP specific) than participants with grade I (€414.4). Müller et al. presented a study in 2019 in which they compared the therapeutic and economic effects of a multimodal back exercise programme^39^. Participants with GCPS grade IV (€5,310) had 2.2 times higher overall direct healthcare costs than participants with grade I (€2,391) over a two-year period.

In this study, which focused only on direct health costs, we also saw a sharp increase in specific BP costs based on GCPS grade. Participants with grade IV (€2,321.12) had about 3.5 higher direct healthcare costs specific for BP than participants with grade I (€650.53). We further identified 1.8 times higher overall direct health costs (€10,619 vs. €5,968) in the last 12 months. The total direct costs of the privately insured were, a lot higher than the costs in the SHI system. This can be explained by the fact that reimbursement schemes and provider spending in the outpatient setting tend to be two to three times higher in the PHI setting^84,85^.

However, this presented study gives a good overview and updated cost information on direct overall and BP-specific costs based on their different GCPS grades. As the relationship of the cost differences between different GCPS grades was in accordance with previous studies, it can be assumed that the figures presented are a good representation of the costs to be expected in the PHI system. Decision makers should use these findings to match effective interventions with limited funds.

### Generalisability

The presented study had three strengths that played a role in the generalisability of the results: I: study sample, II: target group, and III: availability of the data. Due to the long seven-year period in which data were collected, the study reached a large size with 3,506 participants. Furthermore, only data from participants who felt their BP was so pressing that they were willing to participate in an intervention was used. Therefore, the monetary key figures shown reflect the expected costs of people participating in an intervention against their BP.

Another advantage was the availability of the data. To participate, it was mandatory to perform the digital assessment, so that a lot of information could be obtained about BP and its consequences. Furthermore, the cost data collected routinely from the insurance company could be used purposefully. With the exception of the 123 participants who did not provide any invoices in the 12 months prior to enrolment, we were able to use all other available data, as the process to collect patient-reported outcomes prevented missing/incomplete data.

Due to the setting in a PHI, the generalisability of the presented cost data is nevertheless limited. All participants were insured with the same PHI company. Although recruitment took place nationwide, outpatient cost data, and therefore also the overall cost, was probably still higher than could be expected in a comparable study focused on the SHI. This was due to systemic differences between SHI and PHI and cannot be remedied. With knowledge of the two to three times higher costs in the outpatient sector, trends could, albeit also, be gained for the SHI. The trend in the spending between different GCPS grades was, however, highly comparable between PHI and SHI. Furthermore, hospital spending was highly comparable between both systems, as additional private elective benefits, such as supplementation for treatment by a principal physician or accommodation in a single room, were excluded from the cost consideration. Furthermore, the inpatient reimbursement scheme for diagnosis-related groups (DRG) is identical in both systems.

It could also be possible that in an SHI setting, with complete data on coverage against sick leave, more than 70% of the participants would be correctly categorised by the CC. However, the sensitivity analyses with the insured sick leave subgroup showed that the predictive ability only improved slightly. The strength of correlation still stayed in the range of a *fair* agreement between CC and GCPS, so that the influence of the system here could be regarded as low. Overall, the strengths of the study outweigh the inherent disadvantages of PHI claims data so that the results can be interpreted and transferred to other settings. If the classification algorithm of Freytag et al. is used in other settings, care should be taken to ensure that information on diagnoses and medications, as well as work absences due to BP and its duration, is available and reliable.

## Conclusions

This was the first study to analyse the criterion validity of the CC. Sensitivity of 54.6% and specificity of 76.4% of the severity predicted of BP by claims data (CC) with the actual severity of BP by GCPS were determined. In total, 69.7% were correctly classified. With an MCC of 0.304, the strength of correlation was classified as *fair*. Based on the findings of this study, the use of CC as a single tool to determine who is treated against CLBP and with what is not recommended. Healthcare spending can be clearly separated by GCPS. A classification algorithm that could eliminate the need for medical screening on site would need to reach a very high sensitivity to identify patients who would profit the most from a targeted intervention (GCPS grades III and IV). The CC from Freytag could be used to segment the customer engagement approach from the payer's perspective. However, it cannot replace a medical screening instrument. The rate of false negatives would be too high for that.

## Supplementary Information

Supplementary Information.

## Data Availability

The data and code supporting the findings of this study are available from Generali Deutschland Krankenversicherung AG, but restrictions apply to the availability of these data, which were used under license for the current study and are therefore not publicly available. However, they are available from the authors upon reasonable request and with permission including a signed data access agreement of Generali Deutschland Krankenversicherung AG.

## Abbreviations

BP: Back pain
CC: Chronicity class
CCI: Charlson’s comorbidity index score
CI: Confidence intervals
CLBP: Chronic lower back pain
DRG: Diagnosis-related groups
FN: False negative were actual severe BP cases that were wrongly predicted as non-severe
FP: False positive were actual non-severe BP cases that were wrongly predicted as severe
GCPS: Graded chronic pain scale
GP: General practitioner
ICD-10: 10Th revision of the International statistical classification of diseases and related health problems
IQR: Interquartile range
LBP: Low back pain
MCC: Matthews correlation coefficient
PHI: Private health insurance
PHQ-4: Patient health questionnaire 4
SHI: Statutory health insurance
TN: True negative were actual cases of non-severe BP that were correctly predicted as non-severe
TP: True positive were actual severe BP cases that were correctly predicted as severe
