# Supplementary material for: A cross-sectional study to validate an administrative back pain severity classification tool based on the graded chronic pain scale
Source: Sci Rep. 2022 Oct 8;12:16927. doi: 10.1038/s41598-022-21422-x (PMC9547910; doi:10.1038/s41598-022-21422-x)
Supplement: Supplementary file 1 — Supplementary Information. [file 41598_2022_21422_MOESM1_ESM.docx]

## Supplementary material

| **Supplementary Table S1 - Characteristics of study participants based on Chronicity Classes** | | | | | | |
| --- | --- | --- | --- | --- | --- | --- |
|  | **Overall** | ***CC 1*** | **CC 2** | ***CC 3*** | **Self-selected** | **P** |
| N | 3506 | 1756  (50) | 428  (12.2) | 519  (14.8) | 803  (22.9) |  |
| Sex = Female (%) | 1196 (34.1) | 614 (34.9) | 176 (41.1) | 142 (27.4) | 264 (32.9) | <0.001 |
| Age (mean (SD)) | 54.73 (9.52) | 55.12 (9.89) | 53.04 (7.91) | 54.09 (8.03) | 55.22 (10.22) | <0.001 |
| CCI – score (mean (SD)) | 0.80 (1.41) | 0.64 (1.24) | 0.83 (1.29) | 1.08 (1.66) | 0.95 (1.58) | <0.001 |
| Overall health (%) |  |  |  |  |  | <0.001 |
| very good | 49 (1.4) | 34 (1.9) | 5 (1.2) | 5 (1.0) | 5 (0.6) |  |
| good | 505 (14.4) | 360 (20.5) | 41 (9.6) | 39 (7.5) | 65 (8.1) |  |
| moderate | 1832 (52.2) | 962 (54.8) | 204 (47.7) | 242 (46.6) | 424 (52.8) |  |
| bad | 970 (27.7) | 370 (21.1) | 145 (33.9) | 197 (38.0) | 258 (32.1) |  |
| very bad | 150 (4.3) | 30 (1.7) | 33 (7.7) | 36 (6.9) | 51 (6.4) |  |
| PHQ-4 sum score (mean (SD)) | 2.82 (2.56) | 2.38 (2.27) | **3.78 (2.95**) | 3.01 (2.70) | 3.13 (2.64) | <0.001 |
| PHQ-4 subscale depression | 1.56 (1.41) | 1.34 (1.29) | 1.92 (1.54) | 1.67 (1.48) | 1.76 (1.46) | <0.001 |
| PHQ-4 subscale anxiety | 1.26 (1.37) | 1.05 (1.21) | 1.86 (1.62) | 1.33 (1.41) | 1.37 (1.42) | <0.001 |
| Average pain intensity within the last six months (mean (SD)) | 4.48 (1.95) | 4.27 (1.92) | 4.35 (1.83) | 4.84 (2.17) | 4.78 (1.89) | <0.001 |
| Average disability within the last six months (mean (SD)) | 3.99 (2.44) | 3.48 (2.30) | 3.90 (2.28) | 4.98 (2.69) | 4.49 (2.33) | <0.001 |
| Days disabled (%) |  |  |  |  |  | <0.001 |
| 0 - 6 days | 2160 (61.6) | 1305 (74.3) | 223 (52.1) | 168 (32.4) | 464 (57.8) |  |
| 07 - 14 days | 445 (12.7) | 204 (11.6) | 62 (14.5) | 68 (13.1) | 111 (13.8) |  |
| 15 - 30 days | 398 (11.3) | 140 (8.0) | 71 (16.6) | 82 (15.8) | 105 (13.1) |  |
| 31 - 180 days | 503 (14.3) | 107 (6.1) | 72 (16.8) | 201 (38.7) | 123 (15.3) |  |
| Chronicity Classes = 1: Without evidence of chronicity; 2: Evidence of risk of chronicity; 3: Evidence of chronicity | | | | | | |

| **Supplementary Table S2 - Evaluation of the predicted BP severity with self-reported GCPS grades** | | | | |
| --- | --- | --- | --- | --- |
| Predicted BP severity category | Observed BP severity category | | | |
|  | Severe  (GCPS IV) | Risk of chronification (GCPS III) | Non-severe (GCPS I & II) | Total |
| Severe  (CC 3) | TP: 166 | FP: 102 | FP: 175 | 443 |
| Risk of chronification (CC 2) | FP: 53 | TP: 80 | FP: 217 | 350 |
| Non-severe (CC 1) | FN: 123 | FN: 210 | TN: 1270 | 1604 |
| Total | 342 | 392 | 1662 | 2396 |
| Sensitivity | 48.5 % | | | |
| Specificity | 75.9 % | | | |
| Correctly predicted | 63.3 % | | | |
| MCC | 0.244 | | | |
| Spearman’s rho | 0.343 (CI: 0.307 – 0.378), p<0.001 | | | |
| Cohen’s weighted Kappa | 0.307 (0.268 – 0.346) | | | |
| GCPS = Graded Chronic Pain Grade [23] based on self-questionnaire at enrolment  CC = Chronicity Class [31] based on administrative claims data  TP = True positive were actual severe BP cases that were correctly predicted as severe  TN = True negative were actual non-severe BP cases that were correctly predicted as non-severe  FP = False positive were actual non-severe BP cases that were wrongly predicted as severe  FN = False negative were actual severe BP cases that were incorrectly predicted as non-severe | | | | |

| **Supplementary Table S3 – Sensitivity 3x3 confusion matrix for insured against sick leave** | | | | |
| --- | --- | --- | --- | --- |
| Predicted BP severity category | Observed BP severity category | | | |
|  | Severe  (GCPS IV) | Risk of chronification (GCPS III) | Non-severe (GCPS I & II) | Total |
| Severe  (CC 3) | TP: 99 | FP: 52 | FP: 97 | 248 |
| Risk of chronification (CC 2) | FP: 20 | TP: 31 | FP: 115 | 166 |
| Non-severe (CC 1) | FN: 38 | TN: 76 | TN: 586 | 700 |
| Total | 157 | 159 | 798 | 1114 |
| Sensitivity | 52 % | | | |
| Specificity | 78 % | | | |
| Correctly Predicted | 64.3 % | | | |
| MCC | 0.287 | | | |
| Spearman’s rho | 0.405 (CI: 0.355 – 0.453 p<0.001) | | | |
| Cohen’s weighted Kappa | 0.358 (0.298 – 0.418) | | | |
| GCPS = Graded Chronic Pain Grade [23] based on self-questionnaire at enrolment  CC = Chronicity Class [31] based on administrative claims data  TP = True positive were actual severe BP cases that were correctly predicted as severe  TN = True negative were actual non-severe BP cases that were correctly predicted as non-severe  FP = False positive were actual non-severe BP that were incorrectly predicted as severe  FN = False negative were actual severe BP cases that were incorrectly predicted as non-severe | | | | |

| **Supplementary Table S4 - Healthcare usage and costs in the 12 months before enrolment based on CC** | | | | | | | |
| --- | --- | --- | --- | --- | --- | --- | --- |
|  | **Overall** | ***CC 1*** | **CC 2** | ***CC 3*** | **Self-selected** | **P** | **Skew** |
| N (%) | 3506 | 1756 (50.1) | 428 (12.2) | 519 (14.8) | 803 (22.9) |  |  |
| Insured against sick-leave | 1616  (46.1) | 769 (43.8) | 208 (48.6) | 282 (54.3) | 357 (44.5) | <0.001 |  |
| Sick-leave due to BP (% of insured against sick-leave) | 305 (18.8) | 3 (0.4) | 57 (27.4) | 196 (69.5) | 49 (13.8) | <0.001 |  |
| F-Diagnosis available (%) | 644 (18.4) | 102 (5.8) | **334 (78.0)** | 80 (15.4) | 128 (15.9) | <0.001 |  |
| Amount of F Diagnoses (mean (SD)) | 1.42 4.84) | 0.19 (1.18) | **7.21 (9.36)** | 1.24 (4.86) | 1.12 (4.02) | <0.001 |  |
| Amount of ICD-10 M Diagnoses (mean (SD)) | 4.11 (5.67) | 2.60 (3.24) | 3.97 (4.32) | 8.78 (8.95) | 4.47 (6.03) | <0.001 |  |
| **Total Health**  **Cost** € (mean (SD)) | 7279.78 (8040.56) | 5812.52 (6267.95) | 9698.56 (10992.58) | 10277.41 (9452.25) | 7261.71 (7802.49) | <0.001 |  |
| High-Cost cases (%) | 252 (7.2) | 88 (5.0) | 48 (11.2) | 63 (12.1) | 53 (6.6) | <0.001 |  |
| **BP Total**  Cost € (mean (SD)) | 1082.13 (2298.10) | 663.14 (1228.88) | 970.69 (1665.74) | 2543.11 (4246.84) | 1113.49 (2208.33) | <0.001 |  |
| BP Truncated Cost (mean (SD)) | 751.73 (670.23) | 660.18 (612.65) | 736.53 (635.94) | 978.09 (764.57) | 847.17 (718.86) | <0.001 |  |
| High-Cost cases (%) | 325 (9.3) | 69 (3.9) | 36 (8.4) | 140 (27.0) | 80 (10.0) | <0.001 |  |
| Low-Cost cases (%) | 851 (24.3) | 464 (26.4) | 87 (20.3) | 73 (14.1) | 227 (28.3) | <0.001 |  |
| **BP Inpatient**  Cost – € (mean (SD)) | 340.70 (1822.82) | 96.17 (811.75) | 280.96 (1226.98) | 1295.09 (3739.92) | 290.43 (1587.08) | <0.001 |  |
| High-Cost cases (%) | 256 (7.3) | 49 (2.8) | 36 (8.4) | 118 (22.7) | 53 (6.6) | <0.001 |  |
| Low-Cost cases (%) | 3250(92.7) | 1707 (97.2) | 392 (91.6) | 401 (77.3) | 750 (93.4) | <0.001 |  |
| **BP Outpatient**  Cost – € (mean (SD)) | 741.43 (1047.45) | 566.98 (804.18) | 689.73 (944.13) | 1248.02 (1405.14) | 823.06 (1178.59) | <0.001 |  |
| High-Cost cases (%) | 216 (6.2) | 57 (3.2) | 19 (4.4) | 78 (15.0) | 62 (7.7) | <0.001 |  |
| Low-Cost cases (%) | 863 (24.6) | 468 (26.6) | 94 (22.0) | 73 (14.1) | 228 (28.4) | <0.001 |  |
| Chronicity Classes = 1: Without evidence of chronicity; 2: Evidence of risk of chronicity; 3: Evidence of chronicity  High-Cost Cases were calculated using Tukeys Method with 1,5 * IQR [61]  Truncated mean: Exclusion of high-cost cases and cases who did not submit a BP invoice in the last 12 months prior to enrolment. | | | | | | | |
